# Prevalence, correlates, and mental and physical health burden of cardiovascular disease in older U.S. military veterans

**DOI:** 10.1371/journal.pmen.0000192

**Published:** 2024-12-18

**Authors:** Cailin G. Arechiga, Rick Yang, Robert H. Pietrzak

**Affiliations:** 1 Department of Social and Behavioral Sciences, Yale School of Public Health, New Haven, Connecticut, United States of America; 2 Department of Psychology, Harvard University, Cambridge, Massachusetts, United States of America; 3 U.S. Department of Veterans Affairs National Center for PTSD, VA Connecticut Healthcare System, West Haven, Connecticut, United States of America; 4 Department of Psychiatry, Yale School of Medicine, New Haven, Connecticut, United States of America; CiST College, Pokhara University, NEPAL

## Abstract

Cardiovascular disease (CVD) is one of the leading causes of death in the U.S. and is associated with a range of demographic, military, trauma, and clinical characteristics, as well as physical and mental health conditions. Older military veterans may have an increased risk of CVD due to their advanced age and military experiences. To date, however, the prevalence and health burden of CVD in population-based samples of veterans has not been well characterized. This study aimed to characterize the current prevalence of CVD and its association with sociodemographic, military, trauma, and clinical variables in a large, contemporary, and nationally representative sample of older U.S. veterans. Data were analyzed from a cross-sectional sample of 3,001 older U.S. military veterans (aged 60 and older) who participated in the National Health and Resilience in Veterans Study (NHRVS). Veterans were classified according to lifetime CVD status (CVD or no CVD, i.e., diagnoses by a healthcare professional of heart disease, heart attack, and/or stroke). To determine the association of CVD with health status, a comprehensive range of mental and physical health variables was assessed using validated self-report assessments. A total of 25.5% of veterans reported having been diagnosed with CVD. Greater age, cumulative trauma burden, nicotine use disorder, and diagnoses of hypertension, high cholesterol, and diabetes were associated with CVD. CVD was independently associated with a range of mental (odds ratios [ORs] = 1.53–2.27) and physical (ORs = 1.53–3.43) health conditions. Collectively, the results of this study suggest that one in four older U.S. veterans has report being diagnosed with CVD in their lifetimes. Given the broad range of physical and mental health conditions associated with CVD, these findings highlight the importance of integrated and multimodal prevention and intervention efforts for this population.

## 1. Introduction

Epidemiologic studies consistently identify advanced age as a significant risk factor for cardiovascular disease (CVD). The American Heart Association’s recent update shows that 28.2% of men aged 60–79 and 39.6% of those aged 80 and older have heart disease, while the rates for women are 19.6% and 32.8%, respectively [1]. U.S. veterans, who are on average 20 years older than non-veterans, may face increased CVD risk; yet, nationally representative data on CVD prevalence in this group are scarce.

The long-term effects of military service can impact both physical and mental health, potentially heightening CVD risk among older veterans [2–6]. For instance, one conceptual model suggests that military service, especially combat exposure, acts as a “hidden variable in aging men,” negatively influencing the aging process through trauma exposure, injury severity, and related stressors [6]. A study involving over 150,000 participants from the National Health Interview Study revealed that younger veterans (ages 25–70) reported higher rates of cardiovascular conditions than their older counterparts [5]. Additionally, data from the Health and Retirement Study indicated that veterans were twice as likely as non-veterans to develop heart disease (RR = 2.00, 95% CI: 1.69–2.35) [3].

Previous research identifies age, male gender, and race (Black or non-Hispanic White) as primary CVD risk factors [2, 7]. Key contributors such as diabetes, hypertension, and high cholesterol account for approximately 75% of CVD risk [8]. A 2014 study found higher blood pressure and HbA1c levels in male veterans compared to female veterans [9]. Moreover, high cholesterol is linked to mortality and cardiovascular events. A retrospective cohort study of veterans with diabetes indicated that those with elevated systolic blood pressure had worse cardiovascular outcomes [10]. Sociodemographic factors like marital status and education may also offer protective effects against CVD [11]. However, limited data exist on independent CVD risk factors in population-based samples of older U.S. veterans. This study aims to address this gap by examining how a wide range of sociodemographic, military, trauma, and clinical variables relate to CVD prevalence.

Veterans facing significant trauma or health morbidities may experience increased disability and CVD mortality risk throughout their lives. Combat experiences have been linked to higher rates of psychiatric conditions [12–14]. For example, combat veterans are over three times more likely than noncombat veterans to screen positive for lifetime posttraumatic stress disorder (PTSD) and exhibit higher rates of PTSD, suicidal behavior, and chronic pain, independent of other factors [11]. Traumatic experiences, including military sexual trauma, have been associated with increased risks of PTSD and depression, both of which are known CVD risk factors [15–19]. A 27-year study involving over 3.3 million deaths found that individuals with a history of suicide attempts had a significantly elevated risk of ischemic heart disease, regardless of depression severity [20]. Older veterans from specific war eras, like the Gulf and Vietnam Wars, also have higher depression rates, a known CVD risk factor [21, 22].

Psychiatric effects of military service may also manifest years later. A prospective study of over 1.5 million men and 94,000 women receiving VA healthcare found that mental illnesses increased CVD risk over five years [23]. A systematic review highlighted a positive association between PTSD and CVD risk factors, such as elevated heart rate and obesity [24]. PTSD developed decades post-service has also been linked to increased cardiovascular mortality [25]. Additionally, PTSD and related disorders may arise from CVD or cardiovascular events [26, 27]. Research indicates that acute CVD events could heighten PTSD risk and recurrence of cardiovascular events [28]. Data from the 2009–2010 and 2011–2012 National Health and Nutrition Examination Surveys showed a higher prevalence of suicidal ideation in individuals with CVD, particularly among those with prior heart attacks [29]. Lastly, a CDC study found an elevated suicide risk among patients with heart disease and heart failure [30].

Beyond traditional cardiovascular risk factors like hypertension and diabetes, CVD is associated with other physical health issues, including inflammatory conditions such as asthma and arthritis, as well as chronic pain and sleep disorders. Asthma and arthritis are prevalent comorbidities linked to CVD [31–34]. Chronic pain may also result from CVD or related events. For example, a cohort study found that individuals with intense coronary disorders had four times the odds of chronic pain compared to those without [35]. CVD is also linked to chronic kidney disease (CKD), which may be undiagnosed in many CKD patients and can serve as an independent risk factor for CVD [36, 37]. Additionally, CVD is associated with physical disability; a prospective cohort study found that pre-frailty is linked to increased CVD risk [38]. The connection between CVD and other physical health conditions may be partly mediated by psychiatric morbidities. For instance, PTSD can disrupt sleep and contribute to sleep-related issues [39–41]. However, most studies on CVD and physical health conditions have focused on civilians.

In summary, there is a notable lack of data regarding the prevalence, risk factors, and burden of cardiovascular disease among contemporary older U.S. veterans. This study aimed to address this gap by analyzing data from a nationally representative sample of older veterans to achieve the following aims: (1) characterize the lifetime prevalence of CVD among veterans aged 60 and older; (2) examine sociodemographic, military, trauma, and clinical risk factors associated with self-reported CVD; (3) identify mental health conditions related to self-reported CVD; and (4) assess physical health conditions associated with CVD.

## 2. Methods

### 2.1. Participants and procedure

A total of 3,001 older U.S. veterans (age 60+) participated in the 2019–2020 National Health and Resilience in Veterans Study (NHRVS), which surveyed a nationally representative sample of 4,069 veterans from November 2019 to March 2020 (median completion date: November 21, 2019). Participants were recruited from KnowledgePanel, a probability-based online survey panel operated by Ipsos, a multinational survey research company. Participant households were sampled using the U.S. Postal Service Deliver Sequence File (DSF). Participants were given internet access and computers by Ipsos when necessary. The panel is comprised of over 50,000 households that covers approximately 98% of the U.S. adult population. Panel members who endorsed military service (affirmative response to the question: “*Have you ever served on active duty in the U*.*S*. *Armed Forces*, *Military Reserves*, *or National Guard*?”) were eligible to complete the survey; a total of 7,860 veterans were invited to participate in the study and 4,069 completed it (51.8% participation rate). Of these, a total of 3,001 were age 60 or higher and are the focus of the current study. To permit generalizability of results to the U.S. veteran population, the Ipsos statistical team computed post-stratification weights using the following benchmark distributions of U.S. military veterans from the most contemporaneous (August 2019) Current Veteran Population Supplemental Survey of the U.S. Census Bureau’s American Community Survey: age, gender, race/ethnicity, Census Region, metropolitan status, education, household income, branch of service, and years in service [42]. All participants provided informed consent prior to study participation and the VA Connecticut Health Care System Human Subjects Subcommittee approved this study.

### 2.2. Measures

*Cardiovascular Disease Status*. Response of “yes” to: “Has a doctor or healthcare professional ever told you that you have heart disease; has a doctor or healthcare professional ever told you that you have had a heart attack; has a doctor or healthcare professional ever told you that you have had a stroke?” is indicative of a positive screen for CVD.

*Current PTSD* was assessed using the PTSD Checklist for DSM-5 (PCL-5) [43]; a score ≥33 was indicative of a positive screen for PTSD.

*Current MDD* was assessed using the Patient Health Questionnaire-2 (PHQ-2) [44]; a score ≥3 was indicative of a positive screen for MDD.

*Current GAD* was assessed using the Generalized Anxiety Disorder-2 (GAD-2) [45]; a score ≥ 3 was indicative of a positive screen for GAD.

*Current AUD* assessed using the Alcohol Use Disorder Identification Test (AUDIT) [46]; a score ≥ 8 was indicative of a positive screen for AUD.

*Current DUD* was assessed using the Screen of Drug Use [47]; a response of ≥ 7 days is indicative of a positive screen for DUD; a response of 6 or fewer or a response of ≥ 2 days to the question “How many days in the past 12 months have you used drugs more than you meant to?” is indicative of a positive screen for DUD.

*Lifetime PTSD* was assessed using the PTSD Checklist for DSM-5 (PCL-5) [43]; a score ≥ 33 was indicative of a positive screen for PTSD.

*Lifetime MDD*, *AUD*, *and DUD* were assessed using a modified self-report version of the Mini-International Neuropsychiatric Interview for DSM-5 [48]. Standard DSM-5-based algorithms were used to identify positive screens for these disorders.

*Lifetime nicotine use disorder* was assessed using the Fagerström Test for Nicotine Dependence [49]; a score ≥ 6 was indicative of a positive screen for NUD.

*Current suicidal ideation* was assessed via endorsement of “several days or more” in response to item 9 (“How often have you been bothered by thoughts that you would be better off dead; and/or thoughts of hurting yourself in some way over the past 2 weeks”) on the Patient Health Questionnaire-9 (PHQ-9) [50].

*Lifetime suicide attempt*: Response of “yes” to the item: “Have you ever tried to kill yourself?”

*Physical health conditions* were assessed using a checklist of 18 different medical conditions assessed by the item: “Has a doctor or healthcare professional ever told you that you have any of the following medical conditions?” (e.g., arthritis, cancer, diabetes, kidney disease).

*Activities of daily living (ADL) disability* were assessed by asking: “At the present time do you need help from another person to do the following (e.g., bathe, walk around home or apartment)?” [51].

*Instrumental activities of daily living (IADL) disability* were assessed by asking: “At the present time do you need help from another person to do the following (e.g., pay bills or manage money)?” [51].

*Adverse childhood experiences and trauma exposures* were assessed via a count of potentially traumatic events on the Life Events Checklist for DSM-5 [52] and score on the Adverse Childhood Experiences Questionnaire [53].

*Gambling use disorder* was assessed using the Brief Problem Gambling Screen [54]. A response of “yes” to one or more of the following questions is indicative of a positive screen for problem gambling: “In the past 12 months would you say you have been preoccupied with gambling; have you needed to gamble with larger amounts of money to get the same feeling of excitement; have you often gambled longer, with more money or more frequently than you intended to; made attempts to either cut down, control or stop gambling; borrowed money or sold anything to get money to gamble?”

Insomnia was assessed using the Insomnia Severity Index [55]. Score of 15 or higher are indicative of clinical insomnia and scores of 8–14 of subthreshold insomnia.

### 2.3. Data analysis

This secondary data analysis involved an evaluation of the association between self-reported CVD, and military, sociodemographic, clinical, mental health, and physical health variables in a cross-sectional sample of 3,001 older U.S. veterans. Data analyses proceeded in four steps. First, we computed raw, unweighted frequencies, and weighted prevalences of a composite measure of self-reported CVD [56], as well as heart disease, heart attack, and stroke for veterans aged 60+. Second, we conducted independent-samples t-tests and χ^2^ analyses to compare sociodemographic, military, and health risk (HTN, DB, HC) correlates of CVD. We then conducted a series of multivariable binary logistic regression analyses to identify independent correlates of CVD. Sociodemographic, military, and health risk (HTN, DB, HC) correlates that were associated with CVD at the p<0.05 level in bivariate analyses were included in this model. Odds ratios and 95% confidence intervals were computed to quantify magnitudes of associations between these variables. Third, we conducted a series of multivariable binary logistic regression analyses to examine the relation between CVD, and lifetime and current mental health conditions. To adjust for the effects of potential confounding variables, sociodemographic, military, and health risk (HTN, DB, HC) variables that differed between veterans with and without CVD at the p<0.05 level in bivariate analyses were entered as covariates in these models; analyses of current psychiatric disorders and suicidality variables additionally adjusted for lifetime mental health conditions. Odds ratios and 95% confidence intervals were computed to quantify magnitudes of associations between CVD and mental health conditions. Fourth, we conducted a series of multivariable binary logistic regression analyses to examine the relation between CVD and lifetime physical health outcomes. These analyses adjusted for sociodemographic, military, and health risk (HTN, DB, HC) variables that differed between veterans with and without CVD at the p<0.05 level in bivariate analyses, as well as lifetime mental health conditions. Odds ratios and 95% confidence intervals were computed to quantify magnitudes of associations between CVD and physical health conditions. All data analyses were conducted using SAS 9.4 software. All inferential analyses were weighted (e.g., prevalence estimates, regression analyses) and unweighted for reported raw sample sizes.

## 3. Results

The final sample included 3,001 U.S. military veterans aged 60 years and older (mean age = 73.2; SD = 7.9; range = 60–99), the majority of whom were male (96.1%) and White, non-Hispanic (85.2%). Over one-quarter (N = 757; 25.2%) of the sample self-reported having been diagnosed with CVD. With regard to specific CVD conditions, 19.2% (N = 582) reported having been diagnosed with heart disease, 10.1% (N = 290) with a heart attack, and 4.5% (N = 138) with a stroke. A total 17.2% (N = 523) reported being diagnosed with one of these conditions, 7.5% (N = 215) with two conditions, and 0.5% (N = 19) with all three conditions.

**Table 1** shows the demographic, military, and clinical characteristics by self-reported CVD status. Relative to veterans without CVD, veterans with CVD were older, and more likely to identify as male and have an annual household income of <$60,000. They also reported experiencing more traumatic life events, were more likely to screen positive for a lifetime nicotine use disorder and were more likely to report having been diagnosed with high blood pressure, high cholesterol, and diabetes.

**Table 1:** Demographic, military, and clinical characteristics by self-reported cardiovascular disease status in older U.S. military veterans.

	Cardiovascular Disease N = 757 Weighted 25.2%	No Cardiovascular Disease N = 2,244 Weighted 74.8%	Test of difference (χ^2^ or t)	p	Multivariable analysis comparing Cardiovascular Disease to No Cardiovascular Disease
	Weighted mean (SD) or n (weighted %)	Weighted mean (SD) or n (weighted %)			OR (95%CI)
***Demographic Characteristics***					
Age	75.7 (7.9)	72.4 (7.8)	8.99	< .001	1.06 (1.05–1.08)***
Sex			5.30	0.021	
Male	728 (97.7%)	2,063 (95.6%)			1.17 (0.64–2.14)
Female (ref)	29 (2.3%)	181 (4.4%)			
Race/ethnicity			0.70	0.87	-
Non-Hispanic white	664 (85.7%)	1,933 (85.1%)			
Non-Hispanic black	32 (7.6%)	129 (8.4%)			
Hispanic	38 (3.8%)	120 (3.4%)			
Other	23 (2.8%)	62 (3.1%)			
Education			0.16	0.69	-
Some college or less	412 (67.4%)	1,194 (66.5%)			
College graduate or more	345 (32.6%)	1,050 (33.5%)			
Marital status			0.82	0.36	-
Never married/divorced/separated	220 (27.3%)	620 (25.4%)			
Married/partnered	537 (72.7%)	1,624 (74.6%)			
Annual household income			6.57	0.010	
≤ $60K	360 (49.0%)	964 (43.0%)			
> $60K (ref)	397 (51.0%)	1,280 (57.0%)			0.86 (0.71–1.05)
***Military Characteristics***					
Number of deployments			2.96	0.23	-
No deployments	500 (69.1%)	1,550 (70.7%)			
One deployment	154 (19.0%)	460 (19.9%)			
Two or more deployments	96 (11.9%)	217 (9.4%)			
Enlistment status			2.72	0.26	-
Enlisted	553 (70.3%)	1,606 (72.1%)			
Drafted	127 (20.4%)	352 (17.6%)			
Commissioned	76 (9.3%)	284 (10.4%)			
10+ years in military	248 (30.5%)	719 (30.2%)	0.01	0.90	-
***Clinical Characteristics***					
Adverse childhood experiences	1.2 (1.7)	1.1 (1.6)	0.70	0.48	-
Total traumas	8.6 (7.6)	7.5 (7.6)	3.03	0.002	1.03 (1.01–1.04)***
Lifetime MDD and/or PTSD	125 (15.8%)	322 (15.0%)	0.19	0.66	-
Lifetime AUD and/or DUD	311 (39.2%)	858 (39.5%)	0.02	0.89	-
Lifetime NUD	171 (23.3%)	364 (17.7%)	9.16	0.002	1.39 (1.09–1.77)**
High blood pressure	541 (72.4%)	1,263 (58.3%)	38.00	< .001	1.35 (1.08–1.68)**
High cholesterol	516 (67.5%)	1,122 (50.0%)	56.06	< .001	1.74 (1.40–2.15)***
Diabetes	271 (36.8%)	471 (21.5%)	55.71	< .001	1.86 (1.50–2.29)***

*Note*. MDD = major depressive disorder; PTSD = posttraumatic stress disorder; AUD = alcohol use disorder; DUD = drug use disorder; NUD = nicotine use disorder.

In a multivariable analysis, older age, greater number of traumatic life events, lifetime nicotine use disorder high blood pressure, high cholesterol, and diabetes were independently associated with CVD.

**Table 2** shows the prevalence of mental and physical health conditions of the sample by self-reported CVD status. Relative to veterans without CVD, veterans with CVD were more likely to screen positive for current major depressive, posttraumatic stress, and generalized anxiety disorders, as well as current suicidal ideation. Prevalences of lifetime mental health disorders, and current alcohol and drug use, and gambling disorders did not differ by CVD status. Veterans with CVD were also more likely to report having been diagnosed with arthritis, cancer, chronic pain, kidney disease, sleep disorder, migraine, rheumatoid arthritis, and MCI, dementia, or Alzheimer’s disease. They were also more likely to report ADL and/or IADL disability and current insomnia.

**Table 2:** Mental and physical health conditions by self-reported cardiovascular disease status in older U.S. military veterans.

	Cardiovascular Disease N = 757 Weighted 25.2%	No Cardiovascular Disease N = 2,244 Weighted 74.8%	Bivariate Test of difference (χ^2^ or t)	p	Multivariable analyses comparing Cardiovascular Disease to No Cardiovascular Disease
	N (weighted %)	N (weighted %)			OR (95%CI)
*Lifetime*					
Major depressive disorder	91 (10.0%)	219 (9.0%)	0.51	0.47	1.20 (0.87–1.67)
Posttraumatic stress disorder	58 (6.9%)	154 (6.8%)	0.01	0.92	1.13 (0.75–1.70)
Alcohol use disorder	296 (37.2%)	817 (37.8%)	0.08	0.77	0.96 (0.79–1.17)
Drug use disorder	67 (8.6%)	205 (9.7%)	0.60	0.44	1.08 (0.77–1.52)
Suicide attempt	14 (1.5%)	43 (1.5%)	0.00	0.96	1.19 (0.54–2.63)
*Current*					
Major depressive disorder	52 (7.2%)	79 (3.4%)	15.47	< .001	2.27 (1.50–3.45)***
Posttraumatic stress disorder	37 (4.5%)	64 (2.7%)	4.01	0.045	1.91 (1.12–3.25)*
Generalized anxiety disorder	34 (4.5%)	59 (2.7%)	5.04	0.025	1.75 (1.06–2.89)*
Alcohol use disorder	58 (7.7%)	166 (7.7%)	0.00	0.98	1.24 (0.86–1.77)
Drug use disorder	52 (7.7%)	137 (6.2%)	1.53	0.22	1.65 (1.13–2.42)**
Gambling disorder	40 (6.2%)	82 (4.3%)	3.75	0.053	1.53 (1.01–2.32)*
Suicidal ideation	57 (6.5%)	104 (4.2%)	5.35	0.021	1.54 (1.01–2.35)*
*Lifetime*					
Arthritis	367 (47.3%)	907 (39.8%)	10.43	0.001	1.27 (1.04–1.53)*
Asthma, chronic bronchitis, or COPD	115 (13.4%)	264 (11.4%)	1.83	0.18	1.14 (0.86–1.51)
Cancer	217 (28.7%)	551 (24.0%)	5.25	0.022	1.07 (0.86–1.33)
Chronic pain	229 (27.6%)	475 (20.4%)	13.62	< .001	1.48 (1.18–1.85)**
Liver disease	17 (1.8%)	40 (1.7%)	0.05	0.81	1.32 (0.65–2.69)
Kidney disease	104 (14.4%)	110 (4.4%)	69.93	< .001	3.43 (2.47–4.77)***
Sleep disorder	242 (29.3%)	494 (22.0%)	13.13	< .001	1.51 (1.21–1.88)***
Migraine	61 (6.6%)	115 (4.4%)	4.92	0.027	1.53 (1.02–2.31)*
Osteoporosis or osteopenia	56 (5.8%)	142 (4.5%)	1.74	0.19	1.38 (0.90–2.12)
Rheumatoid arthritis	69 (9.0%)	121 (5.6%)	8.13	0.004	1.56 (1.10–2.22)*
Concussion or traumatic brain injury	43 (5.1%)	104 (4.0%)	1.33	0.25	1.44 (0.92–2.27)
MCI, dementia, or Alzheimer’s disease	23 (3.8%)	34 (1.4%)	13.32	< .001	2.21 (1.23–3.98)**
Any Physical Disability	175 (24.5%)	254 (12.0%)	54.71	< .001	1.95 (1.54–2.49)***
ADL disability	61 (8.8%)	93 (4.4%)	16.38	< .001	1.70 (1.17–2.46)**
IADL disability	163 (22.7%)	234 (11.1%)	49.67	< .001	1.92 (1.49–2.46)***
Insomnia			16.24	< .001	
Subthreshold Insomnia	205 (26.3%)	493 (21.7%)			1.37 (1.10–1.72)**
Clinical Insomnia	80 (8.9%)	133 (5.6%)			2.08 (1.44–3.00)***

*Note*. COPD = chronic obstructive pulmonary disease; MCI = mild cognitive impairment; ADL = activities of daily living; IADL = instrumental activities of daily living.

Analyses of lifetime psychiatric variables are adjusted for age, sex, annual household income, and cumulative lifetime trauma burden, which differed by cardiovascular disease status in bivariate analyses (all p’s<0.05); analyses of current psychiatric disorders, suicide attempt and ideation, and physical health variables are additionally adjusted for lifetime major depressive, posttraumatic stress, alcohol, nicotine, and drug use disorders.

In multivariable analyses, CVD was independently associated with increased odds of current major depressive, posttraumatic stress, and generalized anxiety disorders, gambling disorder, and suicidal ideation, as well as arthritis, chronic pain, kidney disease, sleep disorder, migraine, rheumatoid arthritis, MCI dementia or Alzheimer’s disease, any physical disability, and insomnia.

## 4. Discussion

This study provides nationally representative data on the prevalence and overall health burden associated with CVD in older U.S. veterans. Results revealed that over a quarter of older U.S. veterans (25.2%) reported having been diagnosed with composite, self-reported CVD. Given the high mental and physical health burden of CVD observed in this study, these findings underscore the public health significance of CVD in older U.S. veterans.

CVD was strongly associated with a myriad of demographic and clinical characteristics and mental and physical health conditions in our sample of older U.S. veterans, even after adjusting for potential confounding variables. CVD was associated with older age, greater trauma burden, lifetime nicotine use disorder, and CVD risk factors (i.e., high blood pressure and cholesterol, diabetes). In addition, CVD was associated with almost all of the current mental health conditions (e.g., major depressive disorder, PTSD, generalized anxiety disorder) assessed in this study, as well as several potentially debilitating lifetime physical health conditions (e.g., arthritis, chronic pain, sleep disorder) of those considered in this study. Results indicated that study groups did not differ based on several demographic (e.g., race and ethnicity, education, marital status), military (e.g., number of military deployments, enlistment status, years spent in the military), and clinical characteristics (e.g., adverse childhood experiences, lifetime major depressive disorder, and/or PTSD). Although temporality cannot be determined from our cross-sectional study results, these findings are consistent with previous studies, which have shown that CVD is associated with a broad range of demographic and health characteristics among older adults and provide further evidence that this population may particularly benefit from systematic surveillance efforts and timely interventions [57–59].

Greater trauma burden was an independent correlate of CVD in this study. Two pathways may explain this relationship. First, a biological pathway implicating greater traumatic stress and dysregulation of the stress response systems may act as the catalyst for adverse health outcomes such as CVD. For example, traumatic experiences may inundate bodily systems with stress hormones, negatively impact the brain, and increase inflammation, thus leading to adverse health outcomes [60, 61]. Additionally, acute stress induced by traumatic experiences may directly affect heart rate, blood pressure, and influence the onset of cardiac cell death, thus leading to potentially detrimental cardiac events [62]. Second, a behavioral pathway, whereby engaging in risky or poor health behaviors, may link trauma and health outcomes such as CVD [60–62]. Prospective cohort studies of adults with CVD have found that greater trauma exposures may predict unhealthy behaviors such as smoking, nicotine use, tobacco use, or other drug use, unhealthy dietary practices, and physical inactivity, all of which can increase the risk for chronic health conditions–particularly a 38% increased risk for adverse CVD outcomes [63, 64]. These pathways have important clinical relevance for older U.S. veterans, who may experience greater psychological and physical distress due to traumatic exposures during military experience [25–29].

Results of the current study confirm the well-known association between CVD risk factors such as hypertension, high cholesterol, and diabetes, and CVD. They extend this link to older U.S. veterans and underscore the importance of targeting these risk factors as part of primary and secondary prevention and treatment efforts in this population. Previous studies have demonstrated that individuals with underlying health conditions such as diabetes have an increased risk for CVD. For example, a matched case-control study of veterans with diabetes demonstrated that older patients have an increased risk for developing diabetes, with CVD as a complication of the condition in approximately 50% of cases [65]. Given that diabetes may present without symptoms initially, the prevalence could stem from factors such as inadequate diabetes management, delayed diagnosis, limited access to screening, or varying quality of care [65]. Moreover, because diabetes is associated with a higher risk for CVD, it may further be exacerbated by hypertension due to inflammation, insulin resistance, and activation of the immune system [66]. Across all ages and age groups, hypertension has been shown to have an independent association with CVD events such as stroke, myocardial infarction, heart failure, or even sudden death [67]. It is therefore urgent that older veterans with hypertension be screened, monitored, and have access to life-saving care. High cholesterol may also play an influential role in CVD in older veterans and may be attributed to Western lifestyle and dietary patterns. This may result in an increase in plasma accumulation in the arteries, lesions, and plaque, which may cause coronary heart disease and ischemic stroke [68]. Consequently, these clinical characteristics may require greater health care utilization and generate higher health care costs.

Results of this study also suggest that CVD was associated with an elevated likelihood of several current mental health conditions and suicidal ideation. For example, even after conservative adjustment for demographic and trauma characteristics, as well as lifetime mental health conditions, CVD was associated with a 2-fold greater likelihood of screening positive for major depressive disorder (MDD) and a nearly 2-fold greater likelihood for PTSD and generalized anxiety disorder (GAD). In addition, our study found no association between CVD and lifetime mental health conditions, thus suggesting that CVD may lead to the development and diagnosis of current mental health conditions. These results are consistent with previous studies, which have shown that depression, PTSD, and anxiety may be a direct consequence of a cardiac event (including stroke). European meta-analyses, cross-sectional, and observational studies of older U.S. adults (≥ 71 years) have shown that after a clinical stroke diagnosis, 29.3% of patients had some form of anxiety disorder during the first year and 42.2% of patients experienced depression six months after the event [58, 69]. This may be explained by dissatisfaction with the availability of mental health services, physical or cognitive disability resulting from the stroke, or psychological and financial burdens [58, 69].

Moreover, it is important to consider the bidirectional nature of this association and the possible mechanism of cardio-pathogenesis. Anxiety or panic disorder symptoms may exacerbate underlying coronary disease, overlap with coronary heart disease symptoms, and increase creatinine kinase and intraoperative glucose levels [70]. As mentioned above, behavioral factors such as smoking, nicotine use, and avoidance of physical activity may further contribute to this association [71–73]. Individuals with CVD may develop MDD as a consequence of sedentary behavior, which may occur as a form of maladaptive coping and denial of dealing with CVD; sedentary behavior has been found to be associated with increased risk of CVD and all-cause mortality [74, 75]. The association between CVD and depression may further be explained by the “vascular disease hypothesis,” whereby vascular disease may predispose, precipitate, or perpetuate depressive symptoms in older adults [76]. With regard to PTSD, a recent study revealed that medications may serve as traumatic reminders of previous CVD or stroke events and thus cause aversions toward medication adherence due to feelings of nervousness, anxiousness, and anticipation of future adverse events [77]. In fact, veterans with PTSD have been found to be more likely to report nonadherence to preventative medications [78]. A retrospective cohort study of aging veterans (≥ 55 years) further noted the longitudinal impact of PTSD on incident CVD. Even after adjustment for potential confounders, veterans with late-life PTSD had a 45% increased risk for CVD compared to veterans without late-life PTSD, therefore requiring close monitoring and treatment of these mental health conditions [79].

Results of multivariable analyses further revealed that older veterans who reported having been diagnosed with CVD had more than 50% elevated odds of current gambling disorder and suicidal ideation. The observed link between CVD and gambling disorders aligns with a study that analyzed data on adults aged 55 and older who participated in the National Epidemiologic Survey of Alcohol and Related Conditions (NESARC). In this study, at-risk/problem/pathological gambling (ARPG) was prospectively associated with increased incidence of arteriosclerosis or any heart condition, after controlling for sociodemographic, psychiatric conditions, and substance use covariates [80]. Further, a recent case-control study, the largest of its type conducted to date, found that individuals with a gambling disorder have a higher prevalence of CVD [81]. This association may also be understood in the context of “self-determination” theory (SDT) for gambling motivations. Older individuals may desire autonomy, fulfillment, and increased life satisfaction and thus utilize gambling as a “maladaptive strategy” to address these needs [82]. Additionally, they may view gambling as a recreational outlet or form of entertainment, especially if unable to participate in physically demanding leisure activities due to declining health status [82]. The association between CVD and suicidal ideation in older veterans may be a result of high levels of worry, fear, apprehension about the future, lack of motivation, or ability to concentrate, risk of nonadherence to medications, or physical difficulty participating in rehabilitation programs [83]. This association may further be explained by elevated rates of internalizing psychopathology such as depression, PTSD, and GAD, as well as chronic pain and functional disability observed in veterans with CVD. Indeed, previous studies have found that chronic physical pain associated with CVD may lead to suicidality as a consequence of social isolation, life stress, loss of autonomy and dignity, perceptions of uselessness, increased burden on social networks, and physical impairments [84, 85]. Taken together, these findings highlight the importance of routinely screening and monitoring suicide risk in older veterans with and at risk for CVD.

Relative to veterans who did not report having been diagnosed with CVD, veterans who reported CVD were more likely to report being diagnosed with almost all of the physical health conditions assessed. Multimorbidity has been deemed “endemic” among the older population, particularly those with CVD [86]. More than 70% of adults develop CVD by the age of 70 years old, of which more than 65% develop non-CVD comorbidities [86]. This may be due in part to disease-disease interactions (e.g., chronic kidney disease, hypertension, heart failure), disease-drug interactions (e.g., heart failure and arthritis medications), and drug-drug interactions (e.g., medication for one medical condition weakening another) [86]. The lower observed prevalence of CVD (25%) in the current study may be partly accounted for by lack of or reduced health care access, reluctance to seek or underutilization of medical care, or mismanagement of physical health conditions such as the ones mentioned above. This may present difficulties in disease management for health care providers and patients and therefore require innovative approaches to medical care. Non-CVD multimorbidity has been found to be associated with increased symptoms and symptom burden than CVD comorbidities [87]. Multimorbidity has a negative effect on both physical and mental health, thus underscoring the importance of timely treatment and comprehensive disease management [88, 89].

Another important physical health issue to consider for older veterans with CVD is insomnia and sleep disturbance. Sleep disturbances have been found to be independently associated with worse CVD outcomes, with epidemiological studies suggesting that sleep disorders such as sleep apnea are causally linked to CVD and stroke [90, 91]. Indeed, a recent systematic review and meta-analysis found that individuals with insomnia were at 1.69 times greater risk for the development of myocardial infarction [92]. Moreover, for patients recovering from a cardiac event, sleep disturbances have been associated with poor medication adherence, worse mental health (e.g., anxiety, depression), and an obstacle to rehabilitation efforts [93].

Limitations of this study must be noted. First, while nationally representative, the sample predominantly consisted of older, male, non-Hispanic white veterans, which may limit the generalizability of findings to more demographically diverse samples of veterans. Relatedly, educational level did not differ by CVD status, which may be related in part to a cohort effect, as the majority of the sample was older and had completed less than a college degree. Second, this study was cross-sectional, which does not allow us to make causal inferences about the association between CVD and other health conditions. Emerging work suggests that MDD is potentially causally linked to CVD and associated risk factors (e.g., HTN), and that this association may be mediated by antidepressant use [94]. Third, CVD was assessed using a self-report measure that inquired about health care professional-diagnosed conditions; given a greater reluctance to seek health care services among older men [95], and thus lower likelihood of receiving a diagnosis of CVD (i.e., heart disease), it is possible that the observed prevalence may reflect an underestimate of the population-based burden of CVD. Further studies are needed to corroborate study findings, particularly longitudinal studies that may help determine temporality or causal linkages between CVD and mental and physical health outcomes.

## 5. Conclusion

This study provides novel insights about the prevalence and health burden of CVD in a contemporary, nationally representative sample of older U.S. veterans. The prevalence of CVD (25.2%) observed in our study sample underscores the importance of monitoring and screening of veterans who may have risk factors for CVD. Our finding that CVD is associated with a broad range of mental and physical health conditions in older U.S. veterans highlights the importance and urgency of recognizing CVD as a priority for prevention and treatment efforts in this population. Results of the current study also underscore the importance of screening, monitoring, and treating the high prevalence of risk factors and other health conditions that are concomitant with self-reported CVD in older U.S. veterans. Further research comprised of more diverse samples that employ longitudinal and mechanistic research designs are needed to examine the prevalence of and bidirectional associations of CVD with demographic, military, and clinical characteristics. Additional research is also needed to evaluate the effectiveness of individual-, societal-, and policy-level interventions and prevention strategies in mitigating the physical, mental, and socioeconomic burdens of CVD and related multimorbidities among older veterans and other at-risk populations.

## Supporting information

S1 File Limited dataset used in the current study. (XLSX)
